# A Novel 3D Reconstruction Algorithm of Motion-Blurred CT Image

**DOI:** 10.1155/2020/9324689

**Published:** 2020-06-01

**Authors:** Zhang Jing, Guo Qiang, Han Fang, Li Zhan-Li, Li Hong-An, Sun Yu

**Affiliations:** ^1^Department of Computer Science and Technology, Xi'an University of Science and Technology, Xi'an 710054, China; ^2^Department of Information Science and Technology, Northwest University, Changan District, Xi'an 710127, China; ^3^Department of Nephrology, Affiliated Hospital of Qinghai University, Xining Qinghai 810001, China

## Abstract

The majority of medical workers are eager to obtain realistic and real-time CT 3D reconstruction results. However, autonomous or involuntary motion of patients can cause blurring of CT images. For the 3D reconstruction scene of motion-blurred CT image, this paper consists of two parts: firstly, a GAN image translation network deblurring algorithm is proposed to remove blurred results. This algorithm adopts the clear image to supervise the training process of the blurred image, which creates solutions that are close to the clear image. Secondly, this paper proposes a Marching Cubes (MC) algorithm based on the fusion of golden section and isosurface direction smooth (GI-MC) for 3D reconstruction of CT images. The golden section algorithm is used to calculate the equivalent points and normal vectors, which reduces the calculation numbers from four to one. The isosurface direction smooth algorithm computes the mean value of the normal vector, so as to smooth the direction of all triangular patches in spatial arrangement. The experimental results show that for different blurred angle and blurred amplitude, comparing the results of the Shannon entropy ratio and peak signal-to-noise ratio, our GAN image translation network deblurring algorithm has better restoration than other algorithms. Furthermore, for different types of liver patients, the reconstruction accuracy of our GI-MC algorithm is 9.9%, 7.7%, and 3.9% higher than that of the traditional MC algorithm, Li's algorithm, and Pratomo's algorithm, respectively.

## 1. Introduction

The X-ray Computed Tomography (CT) [1] is an advanced anatomical imaging technology, which provides clear images with resolution less than 0.5 mm. However, CT can only provide 2D images of human organs, and doctors in most cases have to rely on their experience to estimate the size and shape of lesions from multiple 2D images. For most doctors, there is a pressing need of the realistic and real-time medical 3D organ results. The 3D visualization technology can display the 3D shape of human organs [2], assisting doctors to analyze the lesions and surrounding tissues, thus providing anatomical structure information which cannot be obtained via traditional ways. Based on this, the surgical planning of orthomorphia and radiotherapy can be realized, which greatly improves the accuracy of medical diagnosis.

In the process of CT imaging, autonomous or involuntary motion of patients can cause blurring of CT images. The autonomous motion includes unconscious movement or body swing of the elderly or children. The involuntary motion indicates the uncontrollable shake of patients (e.g., patients with Parkinson's Disease [3]). Due to the patient's autonomous or nonautonomous movement, CT images will become blurred, which will affect the accuracy of quantitative analysis diagnosis and treatment. Motion blurring is a process in which the pixel values of an image are linearly superposed along the blur angle and magnitude [4]. And when the gray value of each pixel of CT image changes by motion, as a result, that the image has blurred ghosting in a certain direction.

This paper researches the CT blurred image caused by patient's motion. Sun et al. [5] proposed a method to iteratively estimate and compensate motion during reconstruction. In each iteration, the rigid body motion is analyzed by CT image one by one; meanwhile, the system matrix is updated. Zhang et al. [6] proposed a motion compensating a total variation regularization approach, which conducts motion compensation by using interphase deformation vector fields. The motion compensated results are viewed as a static sequence, of which the regularization function was imposed on. Wang et al. [7] used a sparse motion composition method to obtain an estimation of pulmonary motion which linearly combines the respiratory deformation vector field of training samples, subsequently adopting parametric control points on CT to refine the nonrigid pulmonary deformation. Hernandez et al. [8] computed the cross-correlation between every two successive projection images, which estimated the motion waveform from the projection images. However, these motion compensation methods obtain the patient's motion information by means of device tracking or multiframe merging, without saving effective information by its characteristics. Consequently, it is difficult to be widely used in clinical setting.

To resolve the problem, we propose a GAN image translation network deblurring algorithm, which regards deblurring as a special case of image-to-image translation. Therefore, our GAN image translation network deblurring algorithm does not require CT images with time sequence and can preserve texture details in images. GAN is a semisupervised method to train classifiers, which does not need many labeled training sets. A generator is trained to generate realistic samples, while a discriminator is trained to identify the differences between real samples and generated samples [9].

For the 3D reconstruction scene of motion-blurred CT image, this paper includes two parts: firstly, a GAN image translation network deblurring algorithm is proposed to remove blurred results. This algorithm matches the motion-blurred CT images with the corresponding clear images according to the pixels, and the clear image is used to supervise the training process of the blurred image. Secondly, this paper proposes a Marching Cubes (MC) algorithm [10] based on the fusion of golden section and isosurface direction smooth (GI-MC) for 3D reconstruction of CT images. The golden section algorithm [11] calculates the equivalent points and normal vectors, which reduces the calculation numbers from four to one. The isosurface direction smooth algorithm calculates the mean value of the normal vector, so as to smooth the direction of all triangular patches in spatial arrangement. The experimental results show that for different blurred angle and blurred amplitude, comparing the results of the Shannon entropy ratio [12] and peak signal-to-noise ratio [13], our GAN image translation network deblurring algorithm has better restoration than other algorithms. Furthermore, for different types of liver patients, the reconstruction accuracy of our GI-MC algorithm is 9.9%, 7.7%, and 3.9% higher than that of the traditional MC algorithm [14], Li's algorithm [15] and Pratomo's algorithm [16], respectively.

## 2. Methodologies

### 2.1. Motion Blur Removal for CT Images

For CT imaging system, there are many characteristics that would result in the blurred image, such as ray width, ray interval, patient motion, photon scattering, and system noise. It is a phenomenon of image degradation [17]. Ignoring the influence of other characteristics, this paper only considers the image blurring caused by patient motion.

The blurred image caused by motion is described as follows:

(1) $$\begin{array}{c} I_{b}\left(x,y\right)=P\left(x,y\right)*I_{c}\left(x,y\right)+N, \end{array}$$

where *I*_*b*_(*x*, *y*) denotes the blurred image, (*x*, *y*) represents the image pixel. *P*(*x*, *y*) denotes point spread function [18] which describes the distribution characteristics of image pixels. *P*(*x*, *y*) takes into account the change of angle and amplitude caused by motion. *I*_*c*_(*x*, *y*) represents the clear image, ∗ represents the convolution operation [19], and *N* represents the noise component.

**Figure 1:**
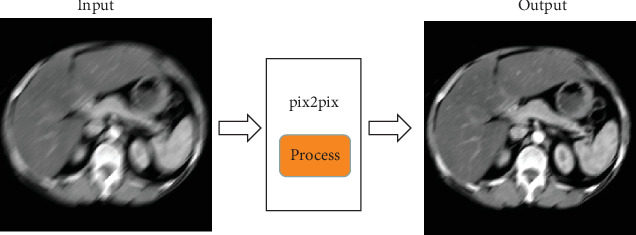
The input and output results of our GAN image translation network.

In order to restore clear CT images from motion-blurred CT images, a GAN image translation network is proposed to remove blurred results. Firstly, the U-net method is used to construct the generator. The idea of skip connection in the U-net method connects the coding network of blurred images with the decoding network of clear images [20], so that the features of the lower sampling layer of the coding network can be transmitted directly to the upper sampling layer of the decoding network, which makes the location of the pixels in the network more accurate. Secondly, the loss functions are selected. To restore clear images from blurred images, both contour information and internal details need to be considered. Therefore, this paper uses the combination of reconstruction loss and adversarial loss. Reconstruction loss is defined by L2 norm [21], which can excellently extract high-frequency information in the image; thus, high-precision image contour is obtained. Adversarial loss can make the generator's internal details closer to the real data distribution [22]. As shown in Figure 1, when the training process of GAN image translation network is completed, the motion-blurred CT image is used as input, and the output result is the clear CT image.

The generative adversarial networks (GAN) is proposed by Goodfellow et al. [23], which consists of two competing parts: the generator and the discriminator. The generator *G* is a network for generating images, which generates images *G*(*z*) by the input *z*. The discriminator *D* is used to judge whether the image *G*(*z*) is “real.” Its input parameter *x* represents an image, and the output *D*(*x*) represents the probability that *x* is a real image. In the training process, the goal of generator *G* is to deceive discriminator *D* by generating convincing perception samples. The goal of discriminator *D* is to separate the generated images by *G* from the real ones. Thus, *G* and *D* constitute a dynamic “game process.” The game between *G* and *D* is the minimax value:

(2) $$\begin{array}{c} \underset{G}{\min}\underset{D}{\max}\underset{x}{E}\left[\log\left(D\left(x\right)\right)\right]+\underset{\overset{\text{\textasciitilde{}}}{x}}{E}\left[\log\left(1-D\left(\overset{\text{\textasciitilde{}}}{x}\right)\right)\right], \end{array}$$

where *E* is the expectation and $\overset{\text{\textasciitilde{}}}{x}=G\left(z\right)$.

#### 2.1.1. Framework

**Figure 2:**
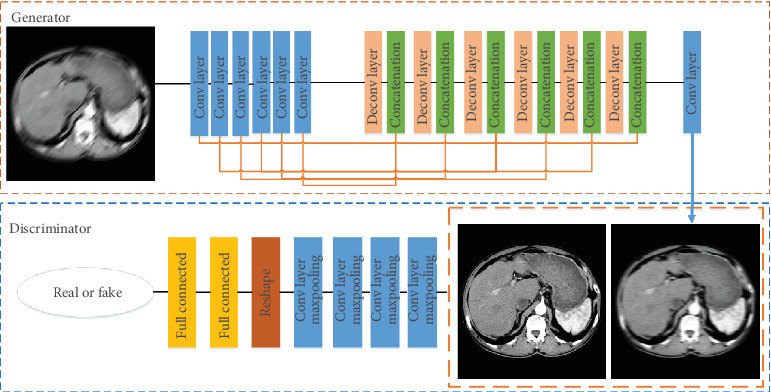
The framework of our GAN image translation network.

The GAN image translation network consists of a generator and a discriminator.  Figure 2 shows the framework of GAN image translation network. In the training process, the generator is responsible for translating CT blurred image into CT clear image, and its input is CT blurred image. The discriminator identifies the difference between the clear image and the output image of the generator by the loss function. The input of the discriminator is the clear image and the output image of the generator.

#### 2.1.2. Network Architecture

*(1) Generator*. It includes the coding network of blurred image and the decoding network of clear image [24]. The idea of skip connection in U-net method is used to connect the coding network of blurred image with the decoding network of clear image.The features in the lower sampling layer of the coding network can be directly transferred to the upper sampling layer of the decoding network, which can make the location of the pixels in the network more accurate. The coding network adopts six convolution layers of C64-C128-C256-C512-C512-C512, where C represents the convolution layer and is represented by the blue box in Figure 2. The decoding network uses six deconvolution layers and one convolution layer of DC512-DC512-DC512-DC256-DC128- DC64-C3, where DC represents the deconvolution layer and is represented by the pink box in Figure 2. The size of each convolution kernel is 5 × 5. The function of concatenation layer is to superimpose the lower sampling layer of the coding network and the upper sampling layer of the decoding network [25], which is represented by the green box in Figure 2. In the convolution and deconvolution layers, the LRelu activation function [26] is used after each layer, while the tanh activation function [27] is used for the clear image reconstruction of the last convolution layer.

*(2) Discriminator*. It is responsible for identifying the real or fake effects of the input blurred images converted into clear images. Our discriminator consists of four convolution layers and two full connection layers. In Figure 2, the convolution layer is represented by the blue box and the full connection layer is represented by the yellow box. The input of the discriminator consists of two parts: the clear image and the output image of the generator. The output of the discriminator is the probability of the similarity between the clear image and the output image of the generator. In the convolution and full connection layer, the LRelu activation function is used after each layer, while the sigmoid activation function [28] is used at the last layer of the full connection layer.

#### 2.1.3. Objective Function

The objective function of this paper consists of reconstruction loss and adversarial loss. The reconstruction loss enables the generator to reconstruct according to the characteristics of the discriminator, and its L2 norm definition can better extract high-frequency information from the image, consequently to obtain high-precision image contours. The adversarial loss matches the generated image according to each pixel and makes the internal details of the generator be closer to the real data distribution. The objective function is defined as follows:

(3) $$\begin{array}{c} L=L_{\text{Reconstruction}}+\lambda L_{\text{adv}}, \end{array}$$

where set *λ* = 0.01 according to the experience value.

*(1) Reconstruction Loss*. We express the pixel-wise Euclidean distance between the generated image and the corresponding clear image as follows:

(4) $$\begin{array}{c} L_{\text{Reconstruction}}\left(I_{s},I_{f}\right)={\left\|G_{\omega }\left(I_{s}\right)-I_{f}\right\|}_{2}^{2}={\left\|\hat{f}-f\right\|}_{2}^{2}, \end{array}$$

where *f* is the ground-truth of the clear image *I*_*f*_ and $\hat{f}$ represents the generated clear image which is the output of input blurred image *I*_*s*_ after the generator *G*_*ω*_, that is $\hat{f}=G_{\omega }\left(I_{s}\right)$.

*(2) Adversarial Loss*. The adversarial loss of our network is defined as follows:

(5) $$\begin{array}{c} L_{\text{adv}}\left(f,\hat{f}\right)=-E\left[\log D_{\psi }\left(f\right)\right]-E\left[\log\left(1-D_{\psi }\left(\hat{f}\right)\right.\right], \end{array}$$

where *f* and $\hat{f}$ have the same meaning in Formula (3). *ψ* represents the parameters of the discriminator networks. *D*_*ψ*_(*f*) and $D_{\psi }\left(\hat{f}\right)$ are the output of the discriminator network. *E* represents the expectation.

### 2.2. GI-MC Algorithm

**Figure 3:**
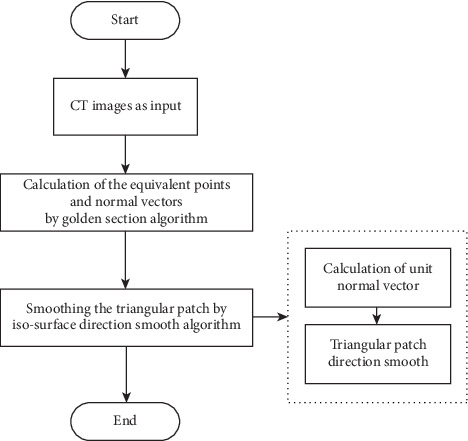
The flow chart of GI-MC algorithm.

To resolve the problems of low computational efficiency and nonsmooth surface of the reconstructed model of the traditional MC algorithm, this paper proposes an MC algorithm based on the fusion of golden section and isosurface direction smooth (GI-MC). Firstly, the golden section algorithm calculates the equivalent points and normal vectors, and the golden section points are used to replace the intersection points of isosurfaces and edges of cubes, which reduce the calculation numbers from four to one. Secondly, due to the discontinuity of normal vectors of triangular patches, there will be a “squamous effect” when displaying the spatial isosurface generated by traditional MC algorithm [29]. In order to get a better visual effect, this paper proposes an isosurface direction smooth algorithm, which smooths all triangular patches that constitute isosurface in spatial arrangement. The flow chart of GI-MC algorithm is shown in Figure 3.

#### 2.2.1. Golden Section Algorithm

When calculating equivalent points and normal vectors, it is complicated when using the traditional linear interpolation algorithm. The edge shared by adjacent cubes needs to be calculated twice, and one edge shared by four cubes needs to be computed four times, which seriously affects the running time. We adopt the golden section algorithm [30] which determine the coordinates and normal vectors of equivalent points through the golden section points of edges.

*(1) Coordinates of Equivalent Points*. If the intersection point is on the *x* axis of the edge, the coordinates of the intersection point are set as $\left(i+\left(\sqrt{5}-1\right)/2,j,k\right)$. If the intersection point is on the *y* axis of the edge, the coordinates of the intersection point are set as$\left(i,j+\left(\sqrt{5}-1\right)/2,k\right)$. If the intersection point is on the *z* axis of the edge, the coordinates of the intersection point are set as $\left(i,j,k+\left(\sqrt{5}-1\right)/2\right)$.

*(2) Normal Vectors of Equivalent Points*. If the intersection point is on the *x* axis of the edge, the normal vector of the intersection point is

(6) $$\begin{array}{c} N=N\left(i,j,k\right)+\left(\left(\sqrt{5}-1\right)/2\right)\left(N\left(i+1,j,k\right)-N\left(i,j,k\right)\right). \end{array}$$

If the intersection point is on the *y* axis of the edge, the normal vector of the intersection point is

(7) $$\begin{array}{c} N=N\left(i,j,k\right)+\left(\left(\sqrt{5}-1\right)/2\right)\left(N\left(i,j+1,k\right)-N\left(i,j,k\right)\right). \end{array}$$

If the intersection point is on the *z* axis of the edge, the normal vector of the intersection point is

(8) $$\begin{array}{c} N=N\left(i,j,k\right)+\left(\left(\sqrt{5}-1\right)/2\right)\left(N\left(i,j,k+1\right)-N\left(i,j,k\right)\right), \end{array}$$

where **N**(*i*, *j*, *k*) represents the vector value of (*i*, *j*, *k*).

#### 2.2.2. Isosurface Direction Smooth Algorithm

Step 1: obtain the coordinate of any triangular patch *t* on the isosurface, which is the 3D coordinate array **P**_*t*_ of three vertex vectors of the triangular patch *t*.

Step 2: calculate the unit normal vector **N**_*t*_ [31] of the triangular patch *t*.

Step 3: triangular patch direction smooth. By smoothing all triangular patches that constitute isosurface in spatial arrangement, the 3D results of the isosurface can be obtained.

*(1) Calculation of Unit Normal Vector*. The data set { (*t*_*i*_, **N**_*t*_*i*__)|*i* = 1, 2, ⋯*M*} of the isosurface is obtained, where *M* is the total number of triangular patches that construct the isosurface and **N**_*t*_*i*__ is the unit normal vector of triangular patch *t*_*i*_. Let **P**_*t*_*i*__ be the 3D coordinate array of three vertex vectors **a**_*i*_, **b**_*i*_, **c**_*i*_ of any triangular patch *t*_*i*_ on the isosurface:

(9) $$\begin{array}{c} P_{t_{i}}=\left[\begin{array}{ccc} x_{a_{i}} & x_{b_{i}} & x_{c_{i}}\\ y_{a_{i}} & y_{b_{i}} & y_{c_{i}}\\ z_{a_{i}} & z_{b_{i}} & z_{c_{i}} \end{array}\right]. \end{array}$$

The unit normal vector **N**_*t*_*i*__ of triangular patch *t*_*i*_ is

(10) $$\begin{array}{c} N_{t_{i}}=R\left(P_{t_{i}}\right)=\frac{\left(b_{i}-a_{i}\right)\times \left(c_{i}-a_{i}\right)}{\left\|\left(b_{i}-a_{i}\right)\times \left(c_{i}-a_{i}\right)\right\|}. \end{array}$$

*(2) Triangular Patch Direction Smooth*. The main idea of triangular patch direction smooth is to smooth the unit normal vector field which constitutes the isosurface. Let the arbitrary triangular patch be *t*_0_, where the triangular patches in the neighborhood of the isosurface are, respectively, *t*_1_, *t*_2_, ⋯, *t*_*n*_. **N**_*t*_1__, **N**_*t*_2__, ⋯, **N**_*t*_*n*__ is the unit normal vector of *t*_1_, *t*_2_, ⋯, *t*_*n*_. After smoothing the direction of the isosurface with the mean of **N**_*t*_1__, **N**_*t*_2__, ⋯, **N**_*t*_*n*__, the unit normal vector **N**_*t*_0__′ of the triangular patch *t*_0_ can be expressed as follows:

(11) $$\begin{array}{c} N_{t_{0}}^{\prime }=\frac{1}{n+1}\overset{n}{\underset{k=0}{\sum }}N_{t_{k}}=\frac{1}{n+1}\overset{n}{\underset{k=0}{\sum }}R\left(P_{t_{k}}\right), \end{array}$$

(12) $$\begin{array}{c} R\left(P_{t_{k}}\right)=\frac{\left(b_{k}-a_{k}\right)\times \left(c_{k}-a_{k}\right)}{\left\|\left(b_{k}-a_{k}\right)\times \left(c_{k}-a_{k}\right)\right\|}, \end{array}$$

where **N**_*t*_*k*__ is the original unit normal vector of the triangular patch *t*_*k*_, **P**_*t*_*k*__ is the original 3D coordinate array of triangular patch *t*_*k*_, and **a**_*k*_, **b**_*k*_, **c**_*k*_ are the three vertex vectors of the triangular patch *t*_*k*_.

By traversing all triangular patches on the original isosurface according to the above algorithm, the corresponding new unit normal vector is {**N**_*t*_*i*__′|*i* = 1, 2, ⋯*M*}. Therefore, the new geometric data set on the isosurface is { (*t*_*i*_, **N**_*t*_*i*__′)|*i* = 1, 2, ⋯*M*}.

## 3. Experiments

In order to evaluate the effect of blurred parameter estimation and motion-blurred restoration more accurately, the motion-blurred image simulated by computer is used in the experiment of this paper. The simulated blurred image is generated on ThinkPad S3-490, of which the processor is Intel® Core™ i5-8265U CPU at 1.60 GHz, and the memory is 8 GB. The algorithm of the simulated blurred image is realized by MATLAB 2018b. The GAN image translation network deblurring algorithm runs on the computer equipped with GeForce RTX 2080Ti GPU and is realized by Python. Our GI-MC algorithm runs in ThinkPad S3-490 for 3D reconstruction of the liver CT image, which is realized by visual studio 2019.

For performance evaluation, we select 2000 CT images of liver from the 3D-IRCADb-01 database [32] (https://www.ircad.fr/research/3d-ircadb-01/). The 3D-IRCADb-01 database is composed of the 3D CT-scans of 10 women and 10 men with liver tumours in 75% of cases. The 20 folders correspond to 20 different patients, which, respectively, contain about 100 images for each anonymized patient in DICOM format. This paper takes the diagnosis results of a liver tumour and a fatty liver patient as examples from the 3D-IRCADb-01 database.

### 3.1. Experiment of Removing Motion Blur

To remove the motion blur of CT image, we mainly research two aspects: (1) identify the blurred angle, which is the direction of motion, and (2) identify the blurred amplitude, which is the amplitude of motion.

#### 3.1.1. Generation of Simulated Blurred Image

The size of the simulated blurred image is 512 × 512 pixels. Because MATLAB has a library function of uniform linear motion, which is the fspecial function. We use the fspecial function to blur the image, and four different experimental parameters are set according to the blurred angle and amplitude.

1. Blurred angle: set the blurred amplitude to 15 pixels, and the range of the blurred angle is 0°, 30°, 60°, and 90°
2. Blurred amplitude: set the blur angle to 45°, and the range of the blurred amplitude is 5, 15, 20, and 25 pixels.

**Figure 4:**
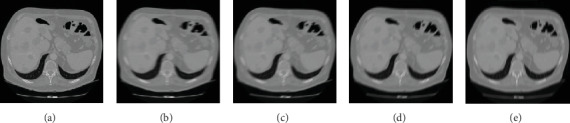
The results of different blurred angle of the liver tumour patient. (a) Original CT image. (b) Blurred angle of 0°. (c) Blurred angle of 30°. (d) Blurred angle of 60°. (e) Blurred angle of 90°.

**Figure 5:**
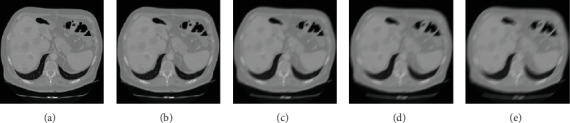
The results of different blurred amplitude of the liver tumour patient. (a) Original CT image. (b) Blurred amplitude of 5. (c) Blurred amplitude of 15. (d) Blurred amplitude of 20. (e) Blurred amplitude of 25.

As shown in Figure 4, (a) is one original CT image of the liver tumour patient. The blurred amplitude was set to 15 pixels. (b) to (e) are the image results with blurred angles of 0°, 30°, 60°, and 90°. As shown in Figure 5, (a) is one original CT image of the liver tumour patient. The blurred angle was set to 45°. (b) to (e) are the image results with blurred amplitudes of 5, 15, 20, and 25 pixels.

**Figure 6:**
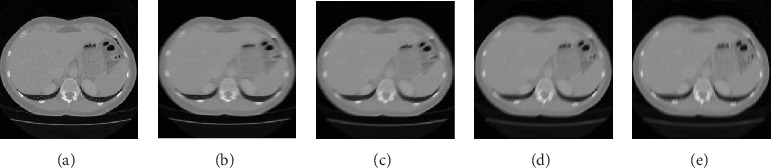
The results of different blurred angle of fatty liver patient. (a) Original CT image. (b) Blurred angle of 0°. (c) Blurred angle of 30°. (d) Blurred angle of 60°. (e) Blurred angle of 90°.

**Figure 7:**
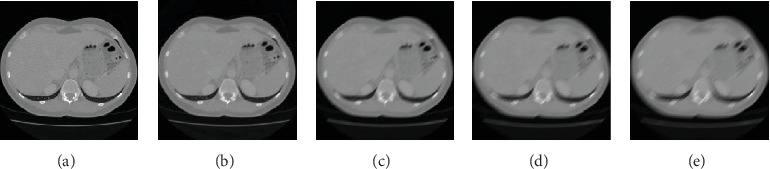
The results of different blurred amplitudes of fatty liver patient. (a) Original CT image. (b) Blurred amplitude of 5. (c) Blurred amplitude of 15. (d) Blurred amplitude of 20. (e) Blurred amplitude of 25.

As shown in Figure 6, (a) is the original CT image of a fatty liver patient. The blurred amplitude was set to 15 pixels. (b) to (e) are the image results with blurred angles of 0°, 30°, 60°, and 90°. As shown in Figure 7, (a) is the original CT image of the fatty liver patient. The blurred angle was set to 45°. (b) to (e) are the image results with blurred amplitudes of 5, 15, 20, and 25 pixels.

#### 3.1.2. Restoration of Blurred Image

**Figure 8:**
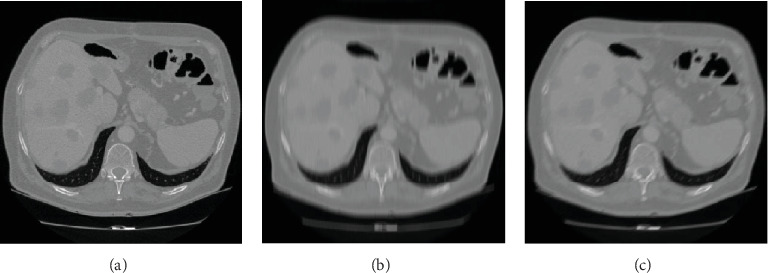
The image restoration results of our algorithm of the liver tumour patient. (a) Clear image. (b) Blurred image. (c) Restored image.

*(1) Qualitative Evaluation*. For the CT image of the liver tumour patient, Figure 8 shows the image restoration results with blurred amplitude of 15 pixels and blurred angle of 90°.  Figure 8(a) is the clear image, and Figure 8(b) shows that the image has ghosting in the moving direction, and the edge of the image is blurred.  Figure 8(c) is the restored image of our algorithm, which found that the blurred edge of the image is well recovered.  Figure 8(c) is very close to Figure 8(a), which shows our algorithm has a good restoration effect and almost restore a clear image.

**Figure 9:**
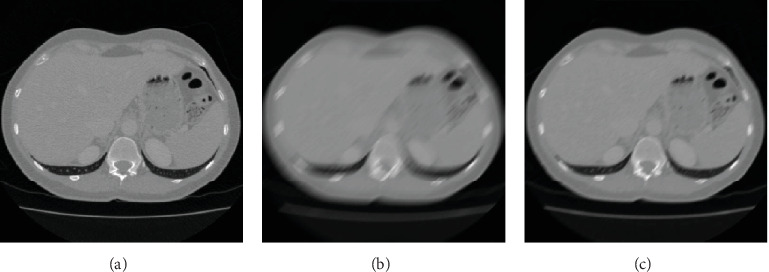
The image restoration results of our algorithm of the fatty liver patient. (a) Clear image. (b) Blurred image. (c) Restored image.

For the CT image of fatty liver patient, Figure 9 shows the image restoration results with blurred angle of 45° and blurred amplitude of 25 pixels.  Figure 9(a) is a clear image.  Figure 9(b) shows that the motion blur makes the volume of the liver larger and the CT image stretched, which will cause errors in the clinical judgment.  Figure 9(c) is the restored image of our algorithm, which found that both the interior and the edge of the image are well restored.

**Figure 10:**
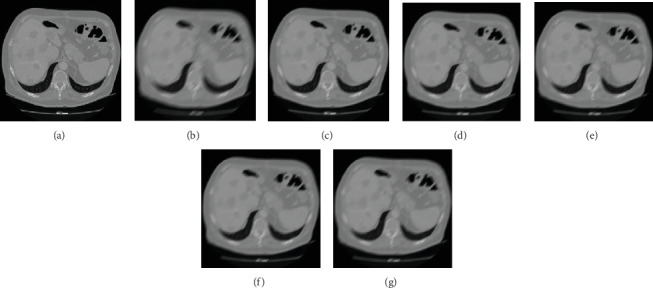
The restored results of various algorithms of the liver tumour patient. (a) Clear image. (b) Blurred image. (c) Our approach. (d) Sun et al.'s algorithm. (e) Zhang et al.'s algorithm. (f) Wang et al.'s algorithm. (g) Hernandez et al.'s algorithm.

For different image deblurring algorithms, our algorithm is compared with Sun et al.'s algorithm [5], Zhang et al.'s algorithm [6], Wang et al.'s algorithm [7], and Hernandez et al.'s algorithm [8].  Figure 10 shows the comparison results of the liver tumour patient of different algorithms with the blurred amplitude of 25 pixels and blurred angle of 45°.  Figure 10(a) is a clear image.  Figure 10(b) is a motion-blurred image with a blurred amplitude of 25 pixels and a blurred angle of 45°.  Figure 10(c) is the restored image of our algorithm.  Figure 10(d) is the restored image of Sun et al.'s algorithm.  Figure 10(e) is the restored image of Zhang et al.'s algorithm.  Figure 10(f) is the restored image of Wang et al.'s algorithm.  Figure 10(g) is the restored image of Hernandez et al.'s algorithm. Compared with other four algorithms, the edge blur of the image in our algorithm is restored better, which is the most similar one to the one of clear image.

**Figure 11:**
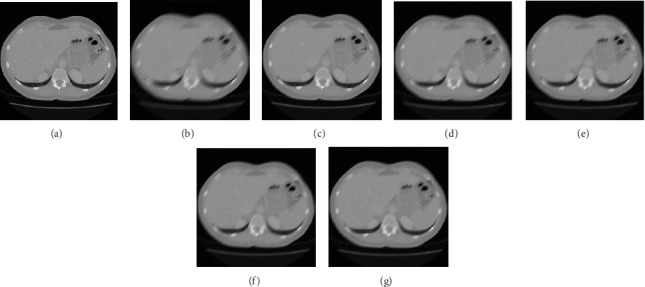
The restored results of various algorithms of fatty liver patient. (a) Clear image. (b) Blurred image. (c) Our approach. (d) Sun et al.'s algorithm. (e) Zhang et al.'s algorithm. (f) Wang et al.'s algorithm. (g) Hernandez et al.'s algorithm.

Figure 11 shows the comparison results of fatty liver patient of different algorithms with the blurred amplitude of 25 pixels and blurred angle of 45°.  Figure 11(a) is a clear image.  Figure 11(b) is a motion-blurred image with a blurred amplitude of 25 pixels and a blurred angle of 45°.  Figure 11(c) is the restored image of our algorithm.  Figure 11(d) is the restored image of Sun et al.'s algorithm.  Figure 11(e) is the restored image of Zhang et al.'s algorithm.  Figure 11(f) is the restored image of Wang et al.'s algorithm.  Figure 11(g) is the restored image of Hernandez et al.'s algorithm. Compared with other four algorithms, both the interior and the edge of the image are better restored.

*(2) Quantitative Evaluation*. We evaluate the effect of CT image restoration using the following two evaluation indicators: the Shannon entropy ratio [12] and the peak signal-noise ratio (PSNR) [13].

(i) *Shannon Entropy Ratio.* Shannon entropy is a method to measure uncertainty. Image restoration will increase the information contained in the image, and the corresponding entropy will be reduced. The Shannon entropy ratio is defined as follows:
  (13) $$\begin{array}{c} E_{r}=\frac{E_{d}}{E_{p}}, \end{array}$$
  where *E*_*d*_ is the Shannon entropy of a blurred CT image or restored CT image and the Shannon entropy of clear CT image. The Shannon entropy is defined as
  (14) $$\begin{array}{c} E=\overset{N}{\underset{i=1}{\sum }}p_{i}\log_{2}\left(1/p_{i}\right), \end{array}$$
  where *N* is the number of histogram groups, and *p*_*i*_ is the frequency of the *i*th histogram of the image.
  (15) $$\begin{array}{c} p_{i}={\text{Num}}_{i}/\left(W*H\right), \end{array}$$
  where *W* is the width of the image, *H* is the height of the image, and *Num*_*i*_ is the number of each histogram in the image. When *p*_*i*_ = 0, 0 × log_2_(1/0) ≡ 0 is set.
(ii) *Peak Signal-Noise Ratio (PSNR).* The PSNR is a statistical analysis indicator based on the gray value of image pixels, which is defined by the mean square error (MSE) between the original image *I*(*i*, *j*) and the restored image *K*(*i*, *j*). Generally, the higher the PSNR value, the better the image restoration.
  (16) $$\begin{array}{c} \text{PSNR}=10\times \log_{10}\left(\frac{{\left(2^{n}-1\right)}^{2}}{\text{MSE}}\right), \end{array}$$
  (17) $$\begin{array}{c} \text{MSE}=\frac{1}{mn}\overset{m}{\underset{i=0}{\sum }}\overset{n}{\underset{j=0}{\sum }}{\left\|I\left(i,j\right)-K\left(i,j\right)\right\|}^{2}. \end{array}$$

We use the Shannon entropy to describe the clarity of the image. The process of image deblurring will increase the information contained in the image, and the corresponding entropy will be reduced. For the CT images of the liver tumour patient, the results of the Shannon entropy ratio of our algorithm, Sun et al.'s algorithm, Zhang et al.'s algorithm, Wang et al.'s algorithm, and Hernandez et al.'s algorithm are shown in Table 1. It can be seen that the Shannon entropy ratio of the restored image is lower than the blurred image before restoration. With the increase of the blurred amplitude, the Shannon entropy ratio of the blurred image will increase, and the Shannon entropy ratio of the restored image will increase. With the increase of the blurred angle, the Shannon entropy ratio of the blurred image and the restored image also increase. The Shannon entropy ratio of our algorithm is smaller than the other four representative algorithms, which shows that our algorithm has the best clarity.

For the CT images of the fatty liver patient, the results of the Shannon entropy ratio of our algorithm, Sun et al.'s algorithm, Zhang et al.'s algorithm, Wang et al.'s algorithm, and Hernandez et al.'s algorithm are shown in Table 2.With the increase of the blurred amplitude, the Shannon entropy ratio of the blurred image will increase, and the Shannon entropy ratio of the restored image will increase. With the increase of the blurred angle, the Shannon entropy ratio of the blurred image and the restored image also increase. For different blurred amplitudes and blurred angles, the Shannon entropy ratio of our algorithm is smaller than the other four representative algorithms.

**Figure 12:**
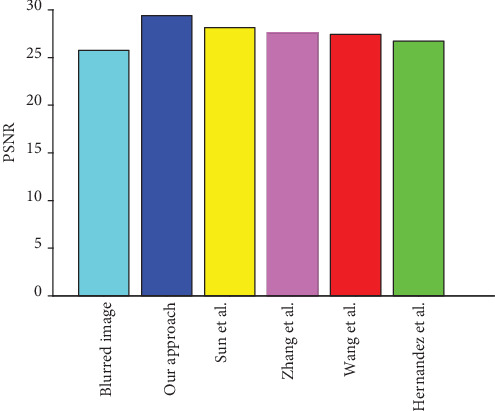
The comparison of PSNR value of the liver tumour patient.

For the CT images of the liver tumour patient, when the blurred amplitude is 25 pixels and blurred angle is 45°, Figure 12 shows the PSNR value result of the blurred image, our algorithm, and four other representative algorithms. The PSNR value of the blurred image is 25.76, our algorithm is 29.39, Sun et al.'s algorithm is 28.13, Zhang et al.'s algorithm is 27.62, Wang et al.'s algorithm is 27.42, and Hernandez et al.'s algorithm is 26.73. It can be seen that the PSNR value of our algorithms is greater than the other four representative algorithms, which achieves a better restoration effect.

**Figure 13:**
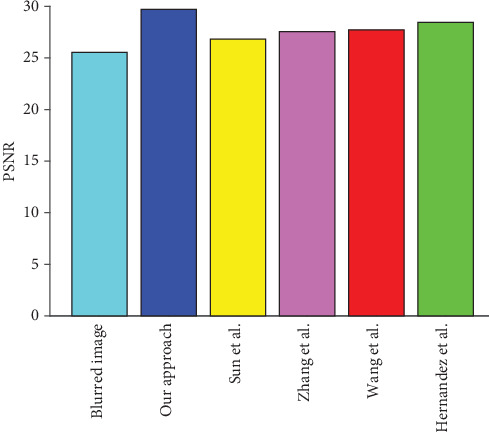
The comparison of PSNR value of fatty liver patient.

For the CT images of fatty liver patient, when the blurred amplitude is 25 pixels and the blurred angle is 45°, Figure 13 shows the PSNR value result of the blurred image, our algorithm, and four other representative algorithms. The PSNR value of the blurred image is 25.55, our algorithm is 29.72, Sun et al.'s algorithm is 26.83, Zhang et al.'s algorithm is 27.55, Wang et al.'s algorithm is 27.71, and Hernandez et al.'s algorithm is 28.44. It can be seen that the PSNR value of our algorithm is greater than the other four representative algorithms, which achieves a better restoration effect.

### 3.2. 3D Reconstruction of Liver

The abdominal CT image contains multiple organs: liver, gallbladder, pancreas, spleen, and kidney. Therefore, before 3D reconstruction of liver, it is necessary to segment liver accurately in the abdominal CT image. We use the region seeds growing algorithm [33] and the histogram threshold [34] method to realize the segmentation of the liver in CT image. The region seeds growing algorithm can roughly determine the location of the liver and avoid mistakenly segmenting other organs. The histogram threshold method can determine the gray value range of liver and segment it accurately. After the accurate segmentation of the abdominal CT image, different algorithms can be used for 3D reconstruction.

We use the time and accuracy of 3D reconstruction as the evaluation indicators to evaluate the performance of different 3D reconstruction algorithms. The comparison algorithms mainly include the traditional MC algorithm [14], Li's algorithm [15], Pratomo's algorithm [16], and our GI-MC algorithm. Li's algorithm is a typical mesh simplification algorithm, which is a representative algorithm for speeding up 3D reconstruction. Pratomo's algorithm improves the 3D reconstruction accuracy by the denoising algorithm, which is the representative algorithm to improve the 3D reconstruction accuracy.

3D reconstruction is performed by CT images of liver tumour and fatty liver patients. The size of the CT image is 512 × 512 pixels. 135 CT images are selected for liver tumour patient, and 113 CT images are selected for fatty liver patient. In order to compare the influence of different algorithms on the time and accuracy of 3D reconstruction, we select four reconstruction algorithms mentioned above for the experiments.

#### 3.2.1. Time of 3D Reconstruction

In the time contrast experiment of 3D reconstruction, two groups of CT images of liver tumour and fatty liver patients are selected. The experiment of each group is repeated 3 times, and then the average value of three results is taken for research.

The computation of traditional MC algorithm is too large, which seriously affects the computation time. For the traditional MC algorithm, there are 135 CT images of 512 × 512 of a liver tumour patient, and 511∗511∗134 = 34990214 voxels need to be traversed. There are 113 CT images of 512 × 512 of a fatty liver patient, and 511∗511∗112 = 29245552 voxels need to be traversed.  Table 3 shows the comparison results of 3D reconstruction time using four algorithms. For two groups of liver patients, it can be seen that the time of GI-MC algorithm has obvious advantages, which greatly reduces the time of 3D reconstruction. It is further found that with the increase of CT images, the number of scanning cubes increases, and our GI-MC algorithm also brings a significant increase in speed.

#### 3.2.2. Accuracy of 3D Reconstruction

The accuracy of 3D reconstruction is the proportion of the liver 3D reconstruction area to the real liver area. To evaluate the results of liver 3D reconstruction, it is necessary to compare with the results marked manually by doctors.

**Figure 14:**
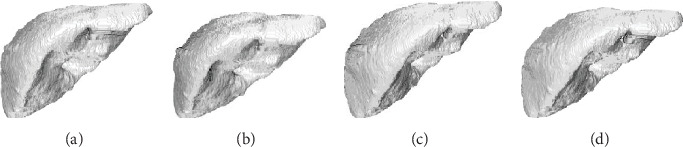
The 3D reconstruction results of four algorithms of the liver tumour patient. (a) Traditional MC algorithm. (b) Li's algorithm. (c) Pratomo's algorithm. (d) Our GI-MC algorithm.

**Figure 15:**
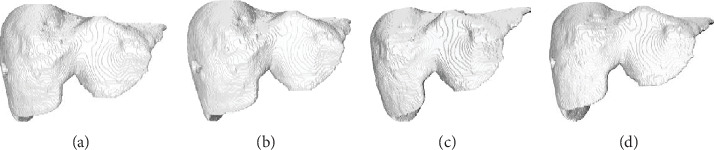
The 3D reconstruction results of four algorithms of the fatty liver patient. (a) Traditional MC algorithm. (b) Li's algorithm. (c) Pratomo's algorithm. (d) Our GI-MC algorithm.

The 3D reconstruction results of four algorithms for CT images of the liver tumour patient are shown in Figure 14. The 3D reconstruction results of four algorithms for CT images of the fatty liver patient are shown in Figure 15. It can be seen that our GI-MC algorithm guarantees the reconstruction quality, the liver surface is smooth, and the texture is fine. There will be a “squamous effect” when displaying the spatial isosurface generated by the traditional MC algorithm. Li's algorithm simplifies the mesh with the idea of edge deletion, but the control of simplified process is difficult. It is easy to lose the details of a small structure. Pratomo's algorithm improves the accuracy of 3D reconstruction by the denoising algorithm, and its reconstruction result has less noise and higher accuracy. Our GI-MC algorithm calculates the mean value of the normal vector of each triangular patch and smooths the direction of all triangular patches in spatial arrangement; thus, the smooth surface of 3D reconstruction of liver is ensured.

The 3D reconstruction results of the four algorithms are compared with the results marked manually by doctors, and then the accuracy of the 3D reconstruction is obtained. From Table 4, the accuracy of the 3D reconstruction of different algorithms can be clearly compared. For the liver tumour patient, compared with the traditional MC algorithm, Li's algorithm, and Pratomo's algorithm, the GI-MC algorithm increases the reconstruction accuracy by 10.4%, 8.1%, and 4.3%, respectively. For the fatty liver patient, compared with the traditional MC algorithm, Li's algorithm, and Pratomo's algorithm, the GI-MC algorithm increases the reconstruction accuracy by 9.4%, 7.3%, and 3.5%, respectively. For data from two groups of liver patients, compared with the traditional MC algorithm, Li's algorithm, and Pratomo's algorithm, the GI-MC algorithm increases by an average of the reconstruction accuracy by 9.9%, 7.7%, and 3.9%.

## 4. Conclusion

In the process of CT imaging, influenced by patient's autonomous or involuntary motion, CT images will be blurred. For the 3D reconstruction scene of motion-blurred CT image, this paper consists of two parts: firstly, a GAN image translation network deblurring algorithm is proposed to remove blurred results. This algorithm adopts the clear image to supervise the training process of the blurred image, which creates solutions that are close to the clear image. Secondly, this paper proposes an MC algorithm based on the fusion of golden section and isosurface direction smooth (GI-MC) for 3D reconstruction of CT images. The golden section algorithm is used to calculate the equivalent points and normal vectors, which reduce the calculation numbers from four to one. The isosurface direction smooth algorithm computes the mean value of the normal vector, so as to smooth the direction of all triangular patches in spatial arrangement. The experimental results show that for different blurred angles and blurred amplitudes, comparing the results of the Shannon entropy ratio and the peak signal-to-noise ratio, our GAN image translation network deblurring algorithm has better restoration than other algorithms. Furthermore, for different types of liver patients, the reconstruction accuracy of our GI-MC algorithm is 9.9%, 7.7%, and 3.9% higher than that of the traditional MC algorithm, Li's algorithm, and Pratomo's algorithm, respectively.

When segmenting the liver region, the traditional manual segmentation method depends on the doctor's experience, which may lead to the actual situation of the operation different from the previous analysis. If we can use the image processing technology and 3D reconstruction technology to accurately segment and reconstruct the abdominal CT images and calculate the volume, the success rate of the operation will be improved. The volume is an important indicator to judge liver disease, and it is also an important basis to decide the operation. In the current clinical treatment, the doctors estimate the volume roughly according to the 2D CT image. Therefore, 3D reconstruction and volume calculation of liver will become a hot topic in the future.

## Figures and Tables

**Table 1:** The Shannon entropy ratio of different algorithms of the liver tumour patient.

Blurred amplitude	Blurred angle	Shannon entropy ratio					
		Blurred image	Our approach	Sun et al.'s algorithm	Zhang et al.'s algorithm	Wang et al.'s algorithm	Hernandez et al.'s algorithm
5 pixels	0°	1.32	1.17	1.22	1.24	1.25	1.27
	30°	1.37	1.21	1.25	1.27	1.28	1.29
	45°	1.39	1.23	1.27	1.29	1.30	1.31
	60°	1.41	1.26	1.30	1.32	1.33	1.35
	90°	1.47	1.31	1.34	1.36	1.37	1.39
25 pixels	0°	1.49	1.22	1.27	1.30	1.31	1.33
	30°	1.54	1.25	1.30	1.31	1.33	1.35
	45°	1.58	1.27	1.33	1.35	1.36	1.39
	60°	1.62	1.31	1.36	1.38	1.39	1.41
	90°	1.67	1.34	1.40	1.42	1.44	1.47

**Table 2:** The Shannon entropy ratio of different algorithms of the fatty liver patient.

Blurred amplitude	Blurred angle	Shannon entropy ratio					
		Blurred image	Our approach	Sun et al.'s algorithm	Zhang et al.'s algorithm	Wang et al.'s algorithm	Hernandez et al.'s algorithm
5 pixels	0°	1.43	1.28	1.38	1.36	1.35	1.33
	30°	1.48	1.32	1.40	1.38	1.37	1.35
	45°	1.51	1.34	1.42	1.40	1.39	1.37
	60°	1.54	1.37	1.45	1.43	1.42	1.40
	90°	1.57	1.41	1.48	1.46	1.45	1.43
25 pixels	0°	1.59	1.33	1.43	1.41	1.40	1.38
	30°	1.63	1.36	1.46	1.44	1.43	1.41
	45°	1.67	1.39	1.48	1.47	1.46	1.44
	60°	1.71	1.42	1.51	1.49	1.48	1.46
	90°	1.77	1.44	1.56	1.54	1.53	1.51

**Table 3:** The comparison results of 3D reconstruction time using four algorithms.

Category	Number of CT images	3D reconstruction time(s)			
		Traditional MC algorithm	Li's algorithm	Pratomo's algorithm	GI-MC algorithm
Liver tumour patient	135	266.97	77.14	71.07	65.93
Fatty liver patient	113	213.62	61.97	56.57	52.27

**Table 4:** The comparison results of 3D reconstruction accuracy of four algorithms.

Category	Number of CT images	3D reconstruction accuracy			
		Traditional MC algorithm	Li's algorithm	Pratomo's algorithm	GI-MC algorithm
Liver tumour patient	135	83.4%	85.7%	89.5%	93.8%
Fatty liver patient	113	82.2%	84.3%	88.1%	91.6%

## Data Availability

For performance evaluation, we select 2000 CT images of liver from the 3D-IRCADb-01 database [32] (https://www.ircad.fr/research/3d-ircadb-01/). The 3D-IRCADb-01 database is composed of the 3D CT-scans of 10 women and 10 men with liver tumours in 75% of cases. The 20 folders correspond to 20 different patients, which, respectively, contain about 100 images for each anonymized patient in DICOM format. [32] G. Pizaine, E. D. Angelini, I. Bloch and S. Makram-Ebeid.Vessel geometry modeling and segmentation using convolution surfaces and an implicit medial axis[C].2011 IEEE International Symposium on Biomedical Imaging: From Nano to Macro, Chicago, IL, 2011 : 1421-1424.
